# Exosome‐shuttled mitochondrial transcription factor A mRNA promotes the osteogenesis of dental pulp stem cells through mitochondrial oxidative phosphorylation activation

**DOI:** 10.1111/cpr.13324

**Published:** 2022-08-26

**Authors:** Jia Guo, Feng Zhou, Zhi Liu, Yuan Cao, Wanming Zhao, Zheru Zhang, Qiming Zhai, Yan Jin, Bei Li, Fang Jin

**Affiliations:** ^1^ Department of Orthodontics, School of Stomatology The Fourth Military Medical University Xi'an China; ^2^ State Key Laboratory of Military Stomatology & National Clinical Research Center for Oral Diseases & Shaanxi International Joint Research Center for Oral Diseases, Center for Tissue Engineering, School of Stomatology The Fourth Military Medical University Xi'an China

## Abstract

**Objectives:**

The treatment of bone defects by stem cells (MSCs) has achieved limited success over the recent few decades. The emergence of exosomes provides a new strategy for bone regeneration. Here, we aimed to investigate the effect and mechanisms of exosomes combined with dental pulp stem cells (DPSCs) on bone regeneration.

**Materials and Methods:**

We isolated exosomes from stem cells from human exfoliated deciduous teeth (SHED) aggregates and evaluated the efficacy of exosomes combined with DPSCs in a cranial bone defect model. The potential mechanisms were further investigated.

**Results:**

The effect of exosomes combined with DPSCs was remarkable on bone regeneration in vivo and exosomes promoted osteogenic differentiation of DPSCs in vitro. Mechanistically, exosomes increased the expression of mitochondrial transcription factor A (TFAM) in DPSCs by transferring TFAM mRNA. Moreover, highly expressed TFAM in DPSCs enhanced glutamate metabolism and oxidative phosphorylation (OXPHOS) activity.

**Conclusions:**

Consequently, exosomes strengthened bone regeneration of DPSCs through the activation of mitochondrial aerobic metabolism. Our study provides a new potential strategy to improve DPSC‐based bone regenerative treatment.

## Graphical abstract

Exosomes increase mitochondrial transcription factor A (TFAM) expression in DPSCs by transferring TFAM mRNA. Moreover, highly expressed TFAM in DPSCs enhances GDH expression and OXPHOS activity. Meanwhile, glutamate metabolism provides amounts of NADH to OXPHOS. Consequently, exosomes strengthen osteogenic differentiation of DPSCs through the activation of mitochondrial aerobic metabolism.

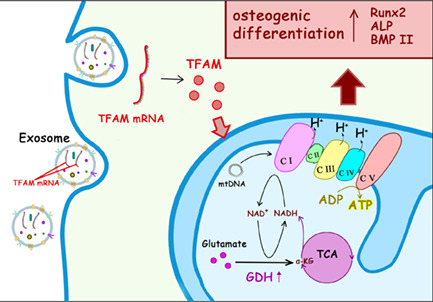

## 1 INTRODUCTION

Bone defect is a problem that needs to be urgently solved in clinical practice. Due to the complexity of the local environment, the lack of autologous bone sources and the difficulties with the blood supply, treatment based on autologous bone grafting is still unable to meet clinical needs. Dental pulp stem cells (DPSCs) located in the pulp tissue of adults have great advantages in bone regeneration, including excellent biological properties, facile obtainment and no serious ethical problems. DPSCs have widely been used in the regeneration of various tissues, including the spinal cord,^1^ pulp,^2^ and long bones.^3^ Thus, DPSC‐based regenerative therapy is expected to replace autologous bone grafting to achieve bone regeneration.

Due to the ability of efficient ATP production to sustain cell function, mitochondria are often considered the “powerhouses of the cell.”^4^ Mitochondria maintain relatively low activity in undifferentiated MSCs and are activated in differentiated MSCs.^5^ Bone marrow stem cells^6^, ^7^ and skull osteoblasts^8^ have been reported to have increased mitochondrial oxidative phosphorylation (OXPHOS) when undergoing osteogenic differentiation. Moreover, both nuclear DNA and mtDNA encode the mitochondrial OXPHOS subunits, where 13 subunits of the OXPHOS complexes are controlled by mtDNA. TFAM, a nuclear‐coding protein transported into mitochondria from the cytoplasm, binds specifically to the mtDNA promoter, recruits RNA polymerase to enable the transcription of mtDNA and thus enhances upregulations of five mitochondrial complexes.^9^ The deficiency of TFAM causes mitochondrial metabolic reprogramming and predominantly depends on glycolysis through the increased activity of lactate dehydrogenase.^10^ Thus, TFAM may be the key to influence osteogenesis through mitochondrial metabolism.

As a newly recognized mechanism of bone formation and bone homeostasis, exosomes shuttle selective proteins, lipids, nucleic acids and glycoconjugates,^11^ which are necessary for cell communication. It has been confirmed that exosomes are important stimulators of the osteogenic differentiation of MSCs.^12^ A previous study demonstrated that SHED‐derived conditioned exosomes enhanced osteogenic differentiation of MSCs in vitro.^13^ In addition, exosomes often play an inducing role in metabolic reprogramming in physiological and pathological activities. For example, oligodendrocytes enhance axonal energy metabolism,^14^ and tumour‐derived exosomes activate glycolysis to promote bone metastasis.^15^ It remains unclear whether exosomes from SHED aggregates promote osteogenic differentiation of DPSCs, and the underlying mechanisms by which exosomes could regulate mitochondrial metabolism require in‐depth exploration.

In the present study, we investigated the effects of DPSCs combined with exosomes derived from SHED aggregates on bone regeneration in a cranial bone defect model in mice and a mandibular bone defect model in rats. Mechanistically, exosomes transferred TFAM mRNA and increased TFAM expression in DPSCs, which activated mitochondrial OXPHOS and osteogenic differentiation. Our research revealed the unrecognized role of exosomes in transferring the mtDNA regulator TFAM to enhance osteogenic differentiation, which provides a novel strategy for the treatment of bone loss.

## 2 RESULTS

### 2.1 Exosomes derived from SHED aggregates promote osteogenic properties of DPSCs

**FIGURE 1:**
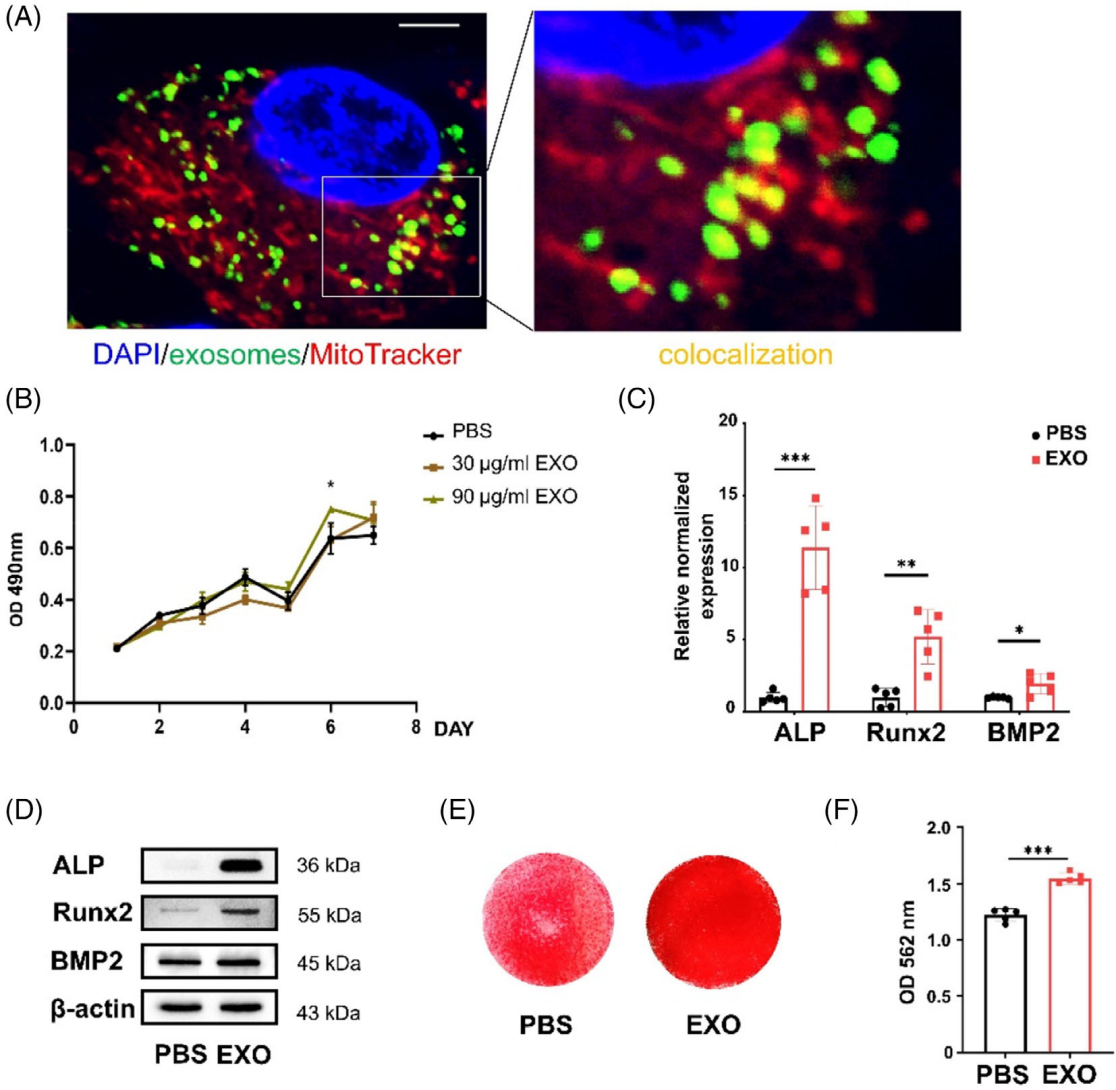
Exosomes promote proliferation and osteogenic differentiation of dental pulp stem cells (DPSCs). (A) Confocal fluorescence analysis showed that exosomes labelled with PKH‐67 (green) were taken up by DPSCs labelled with DAPI (blue) and MitoTracker (red) after 24 h. The confocal area (yellow) exists between exosomes (green) and mitochondria (red). Scale bar: 5 μm. (B) The proliferation of DPSCs treated with different concentrations of exosomes (0, 30, 90 μg/mL). *N* = 5 independent experiments. (C–E) quantitative real‐time reverse transcriptase–polymerase chain reaction (qRT‐PCR) analysis (C) (*N* = 5 independent experiments), western blot analysis (D) and alizarin red staining (E) showed that exosomes promoted osteogenic differentiation of DPSCs. (F) Quantitative analysis of the calcium deposition in DPSCs. *N* = 5 independent experiments. *p < 0.05; **p < 0.01; ***p < 0.001. Error bars are mean ± SD.

Tissue‐specific MSCs in the dental pulp were successfully isolated from healthy adults (DPSCs) and children (SHED) and detected by flow cytometry (Figure S1A–D). Then, exosomes were collected from the supernatant of SHED aggregates and identified by transmission electron microscopy (TEM), nanoparticle tracking analysis (NTA) and western blot analysis. TEM and NTA showed that exosomes exhibited a typical morphology with a sphere‐like bilayer membrane structure (Figure S1E), with a diameter of about 50–150 nm (Figure S1F). Western blot analysis showed that exosomes expressed surface markers, including CD9, CD63, CD81 and TSG101, except for a negative marker Golgin 84 (Figure S1G). To investigate whether exosomes could be internalized into DPSCs, exosomes labelled with PKH67 were incubated with DPSCs for 24 h. Confocal fluorescence microscopy analysis showed not only exosomes (green) internalized by DPSCs but also colocalization between exosomes and mitochondria (MitoTracker‐red) in DPSCs (Figure 1A).

We performed a functional assay to assess whether exosomes derived from SHED aggregates contribute to the osteogenic differentiation of DPSCs. First, the osteogenic effects of exosomes at different concentrations (0, 30, 60, 90, 120 and 150 μg/ml) were assessed by quantitative real‐time reverse transcriptase–polymerase chain reaction (qRT‐PCR). In comparison with groups with other exosomes' concentrations (0, 30, 60, 120 and 150 μg/mL), the expression of Runt‐related transcription factor 2 (Runx2) was significantly increased in the 90 μg/ml exosomes group (Figure S2A). DPSCs treated with 90 μg/ml exosomes showed enhanced proliferation ability compared with those treated with other concentrations of exosomes (0 and 30 μg/ml) after 6 days (Figure 1B). We then evaluated the osteogenic differentiation properties of DPSCs treated with 90 μg/ml exosomes (EXO group) and equal phosphate buffer saline (PBS group). Higher mRNA levels of alkaline phosphatase (ALP), Runx2 and bone morphogenetic protein 2 (BMP2) were found in the EXO group compared with the PBS group as analysed by qRT‐PCR (Figure 1C). Consistently, the EXO group expressed more osteogenic proteins, including ALP, Runx2 and BMP2, than the PBS group (Figure 1D, Figure S2B). The alizarin red staining of the EXO group showed more mineralized nodules than the PBS group (Figure 1E, F). Hence, 90 μg/ml exosomes significantly promoted osteogenic differentiation of DPSCs in vitro, and this dose was selected for the following experiments.

### 2.2 Exosomes enhance DPSC‐mediated repair of cranial and mandibular bone defects

**FIGURE 2:**
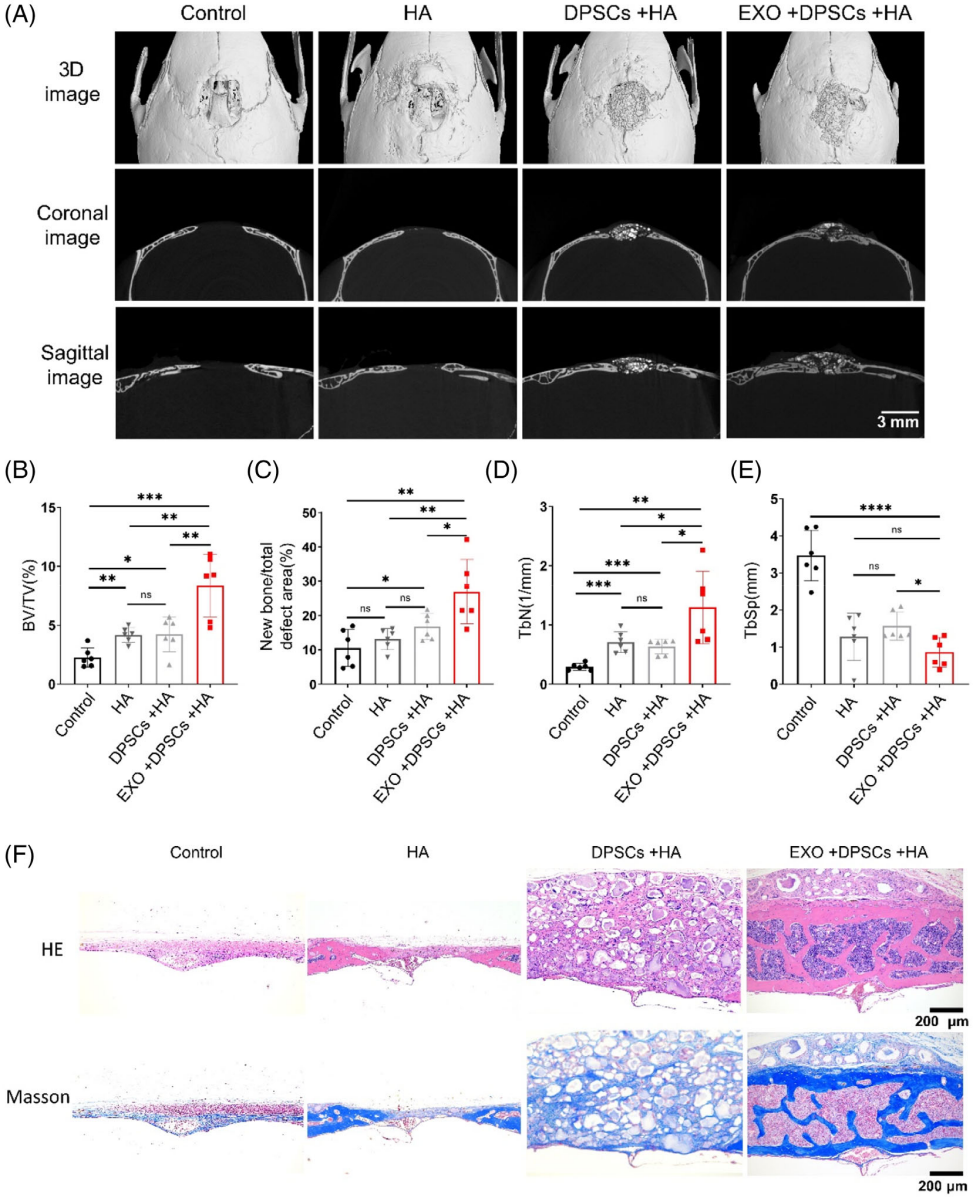
Exosomes (EXO) enhance dental pulp stem cells (DPSCs)‐mediated repair of the cranial bone defect. (A) Micro‐CT sagittal and coronal section images of the Control, hydroxyapatite (HA), DPSCs + HA, EXO + DPSCs + HA groups in the cranial bone defect. Scale bar: 3 mm. (B–E) Quantitative analysis of bone volume/total volume (BV/TV) (B), new bone area/total defect area (C), mean bone trabecula number (Tb.N) (D), and mean trabecula spacing (Tb.Sp) (E). *N* = 6 independent samples. (F) Hematoxylin and eosin (H&E) staining and Masson trichrome staining of coronal sections. Scale bar: 200 μm. *p < 0.05, **p < 0.01, ***p < 0.001, ****p < 0.0001. Error bars are mean ± SD.

To investigate the in vivo bone regeneration capacity of DPSCs with exosomes, we implanted DPSCs combined with exosomes on a model of cranial bone defects in mice. Micro‐computed tomography (micro‐CT) showed bone repair of each group in three dimensions (Figure 2A). Quantitative analysis showed that bone volume/total volume (BV/TV) (Figure 2B), new bone area/total defect area (Figure 2C), and mean bone trabecular number (Tb.N) (Figure 2D) increased, whereas mean trabecular spacing (Tb.Sp) (Figure 2E) in the EXO + DPSCs + HA group significantly decreased compared with the DPSCs + HA group. Moreover, the cranial defects in the control group were filled with fibre structures, and nearly no new bone was observed in haematoxylin–eosin staining (H&E) and Masson staining (Figure 2F). The visible defects in the hydroxyapatite (HA) group were still connected by fibrous structures, but there was a small amount of new bone wrapped in HA particles at the end of the bone plate. Similar to the HA group, the defects in the DPSCs + HA group were filled with implanted DPSCs and HA particles, and there was a small amount of new bone at the ends. The defects in the EXO + DPSCs + HA group were continuous in the bone plate and contained many medullary cavity‐like structures, indicating that bone‐like tissue was regenerated.

In addition, we adopted a rat model of mandibular bone defect. As shown by micro‐CT, there was obvious bone regeneration in the EXO + DPSCs + HA group compared with the Control, HA and DPSCs + HA groups (Figure S2C, D). HE staining and Masson trichrome staining showed increased new bone formation and more collagen deposition in the EXO + DPSCs + HA group (Figure S2E). Therefore, the results indicated that the combination of DPSCs with exosomes increased cranial and mandibular bone formation in vivo.

### 2.3 Exosomes improve the expression of TFAM and enhance OXPHOS in DPSCs

**FIGURE 3:**
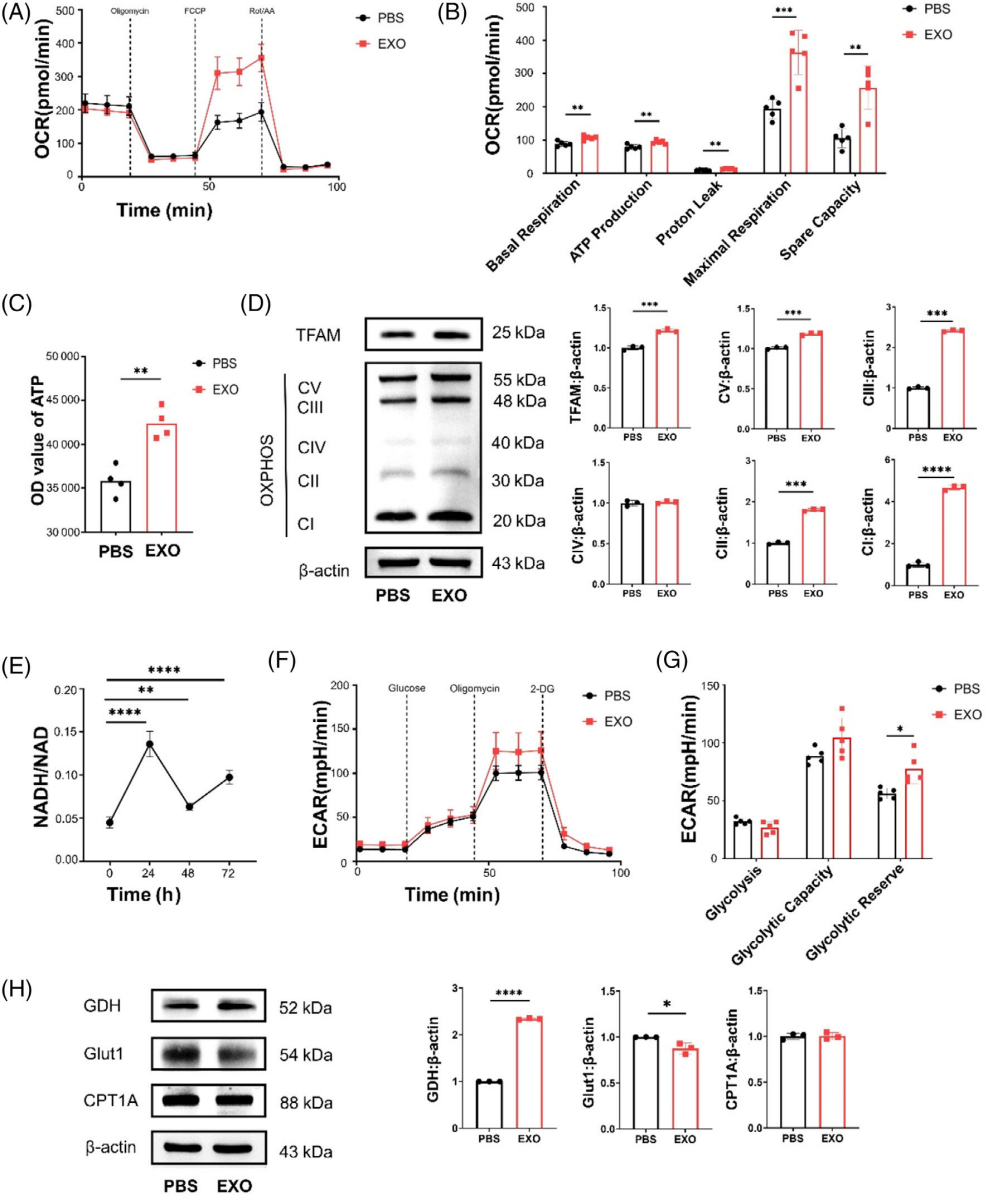
Exosomes improve mitochondrial transcription factor A (TFAM) expression and oxidative phosphorylation (OXPHOS) activity in DPSCs. (A) oxygen consumption rate (OCR) from seahorse analysis in DPSCs with supplementation of exosomes. (B) Basal respiration, ATP production, proton leak, maximal respiration and spare capacity in OCR assay. *N* = 5 independent experiments. (C) The volume of ATP in DPSCs with supplementation of exosomes. *N* = 4 independent experiments. (D) The protein expression of TFAM and five OXPHOS complexes by western blot analysis. (E) NADH/NAD of DPSCs with exosomes for 24, 48 and 72 h. *N* = 4 independent experiments. (F) Extracellular acidification rate (ECAR) from seahorse analysis in DPSCs with supplementation of exosomes. (G) Glycolytic reserve, glycolysis and glycolytic capacity in ECAR assay. *N* = 5 independent experiments. (H) The expression of GDH, Glut1 and CPT1A by western blot analysis. *N* = 3 independent experiments. *p < 0.05; **p < 0.01; ***p < 0.001, ****p < 0.0001. Error bars are mean ± SD.

Based on our observation of colocalization between exosomes and mitochondria in DPSCs (Figure 1A), we assumed that the improvement of osteogenic differentiation was related to the mitochondrial function of DPSCs. Consistent with previous reports, we also found that the oxygen consumption rate (OCR) of DPSCs was increased after osteogenic induction (Figure S3A), as indicated by the increased levels of basal, maximal and ATP production respiration rate and spare respiratory capacity (Figure S3B). Similarly, exosomes exhibited an increased ability to improve mitochondrial OXPHOS of DPSCs (Figure 3A) under the growth mediums, as indicated by the increased levels of basal, maximal and ATP production respiration rate and spare respiratory capacity (Figure 3B). We also confirmed by luminescent assay that the EXO group produced more ATP than the PBS group (Figure 3C). Exosomes enhanced the expression of mitochondrial complexes I–V, as detected by western blot (Figure 3D). As a regulator of mtDNA transcription, TFAM expression analogously increased (Figure 3D). Nicotinamide adenine dinucleotide (NADH) produced by the TCA cycle drives the electronic respiratory chain to produce ATP.^16^ TFAM knockdown could decrease the NADH/NAD ratio.^17^ The NADH/NAD ratio in DPSCs supplemented with exosomes was elevated at 24, 48 and 72 h (Figure 3E).

As exosomes significantly heightened the OCR of DPSCs, DPSCs might consume a certain nutrient increase, such as glucose, amino acids and fatty acids. However, the extracellular acidification rate (ECAR) of glucose metabolism did not differ between the EXO group and the PBS group (Figure 3F), as indicated by the same levels of glycolysis, glycolysis capacity and slightly increased level of glycolysis reserve (Figure 3G). Glutamate dehydrogenase (GDH), mainly localized in the mitochondrial matrix, catalyses oxidative deamination of l‐glutamate to α‐ketoglutarate (α‐KG) into the TCA cycle.^18^ Carnitine palmitoyltransferase (CPT) is a key enzyme of mitochondrial fatty acid oxidation.^19^ Here, we found that exosomes slightly reduced the expression of glucose transporter 1 (Glut1) and increased the expression of GDH, and there was no change in the expression of CPT1A (Figure 3H). To explore whether the mitochondrial function was in a steady state under the action of exosomes, we detected the redox status of DPSCs at 24, 48 and 72 h after adding exosomes. Reactive oxygen species (ROS) level of DPSCs did not change significantly (Figure S3C). Hence, we showed that exosomes promoted TFAM and GDH expression and enhanced mitochondrial OXPHOS in DPSCs.

### 2.4 Exosomes strengthen mitochondrial OXPHOS and osteogenic differentiation of DPSCs through TFAM

**FIGURE 4:**
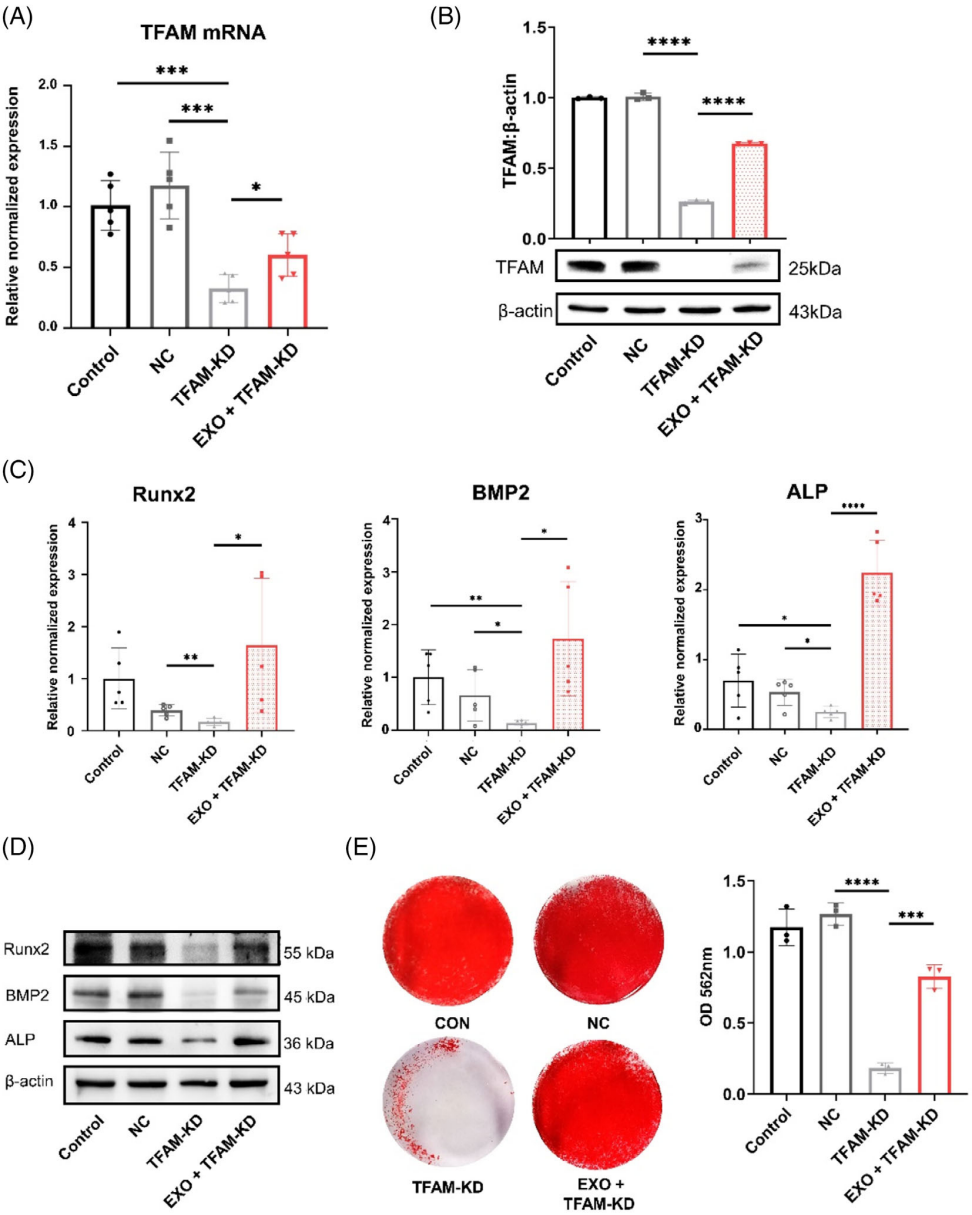
Exosomes (EXO) enhance osteogenic differentiation of dental pulp stem cells (DPSCs) through mitochondrial transcription factor A (TFAM). (A) The TFAM mRNA of the CON, NC, TFAM‐KD, and EXO + TFAM‐KD groups by quantitative real‐time reverse transcriptase–polymerase chain reaction (qRT‐PCR) analysis. *N* = 5 independent experiments. (B) The TFAM protein expression of the CON, NC, TFAM‐KD and EXO + TFAM‐KD groups by western blot analysis. *N* = 3 independent experiments. (C–E) The qRT‐PCR analysis (C) (*N* = 5 independent experiments), western blot analysis (D) (*N* = 3 independent experiments) and alizarin red staining (E) showed that exosomes restored osteogenic differentiation of TFAM‐KD DPSCs. Quantitative analysis of the calcium deposition in DPSCs (*N* = 3 independent experiments). *p < 0.05; **p < 0.01; ***p < 0.001; ****p < 0.0001. Error bars are mean ± SD.

As we observed the augmentation of TFAM mediated by exosomes, we examined whether this effect would enhance mitochondrial OXPHOS to promote osteogenic differentiation of DPSCs. The qRT‐PCR assay and western blot showed that exosomes partly restored TFAM expression in TFAM‐KD DPSCs (Figure 4A, B). Next, we detected the necessity of TFAM expression for osteogenic differentiation. Similarly, qRT‐PCR assay revealed decreased mRNA levels of ALP, Runx2 and BMP2 in TFAM‐KD DPSCs compared with the non‐specific control (NC) group, but the addition of exosomes rescued the decreased expression of these genes (Figure 4C). Consistent with the gene expression, the TFAM‐KD group showed decreased osteogenic proteins, such as ALP, Runx2 and BMP2, compared with the NC group, and the EXO + TFAM‐KD group expressed these proteins more than the TFAM‐KD group (Figures 4D, S3D). The alizarin red staining showed that mineralization of the EXO + TFAM‐KD group repaired the decreased osteogenesis of the TFAM‐KD group (Figure 4E).

**FIGURE 5:**
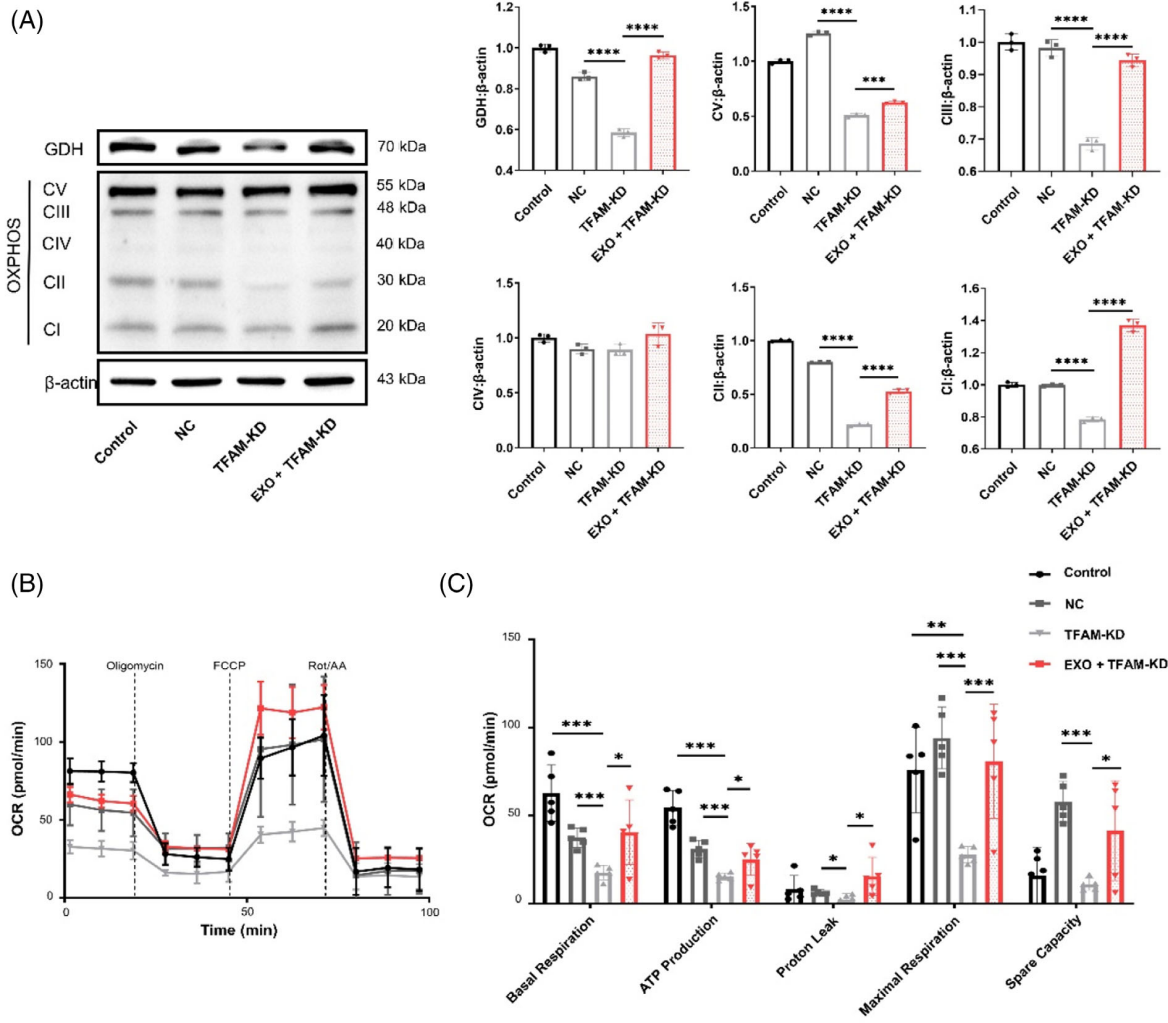
Exosomes strengthen the oxidative phosphorylation (OXPHOS) activity of dental pulp stem cells (DPSCs) through mitochondrial transcription factor A (TFAM). (A) The expression of TFAM and total OXPHOS complexes by western blot analysis. *N* = 3 independent experiments. (B) Oxygen consumption rate (OCR) from seahorse analysis in DPSCs with supplementation of exosomes. (C) Basal respiration, ATP production, proton leak, maximal respiration and spare capacity in OCR assay. *N* = 5 independent experiments. *p < 0.05; **p < 0.01; ***p < 0.001; ****p < 0.0001. Error bars are mean ± SD.

Furthermore, there was a significant decrease in protein expression of GDH, and mitochondrial complexes I–V in TFAM‐KD DPSCs, but the addition of exosomes increased the expression of these proteins (Figure 5A). Interestingly, exosomes restored the decreased mitochondrial respiratory capacity of TFAM‐KD DPSCs (Figure 5B), as indicated by the increased levels of basal, maximal, and ATP production respiration rate and spare respiratory capacity (Figure 5C). Taken together, these results suggest that TFAM is a key point to regulate downstream mitochondrial OXPHOS and promote osteogenic differentiation of TFAM‐KD DPSCs.

### 2.5 Exosomes shuttle TFAM mRNA to heighten osteogenic differentiation of DPSCs

**FIGURE 6:**
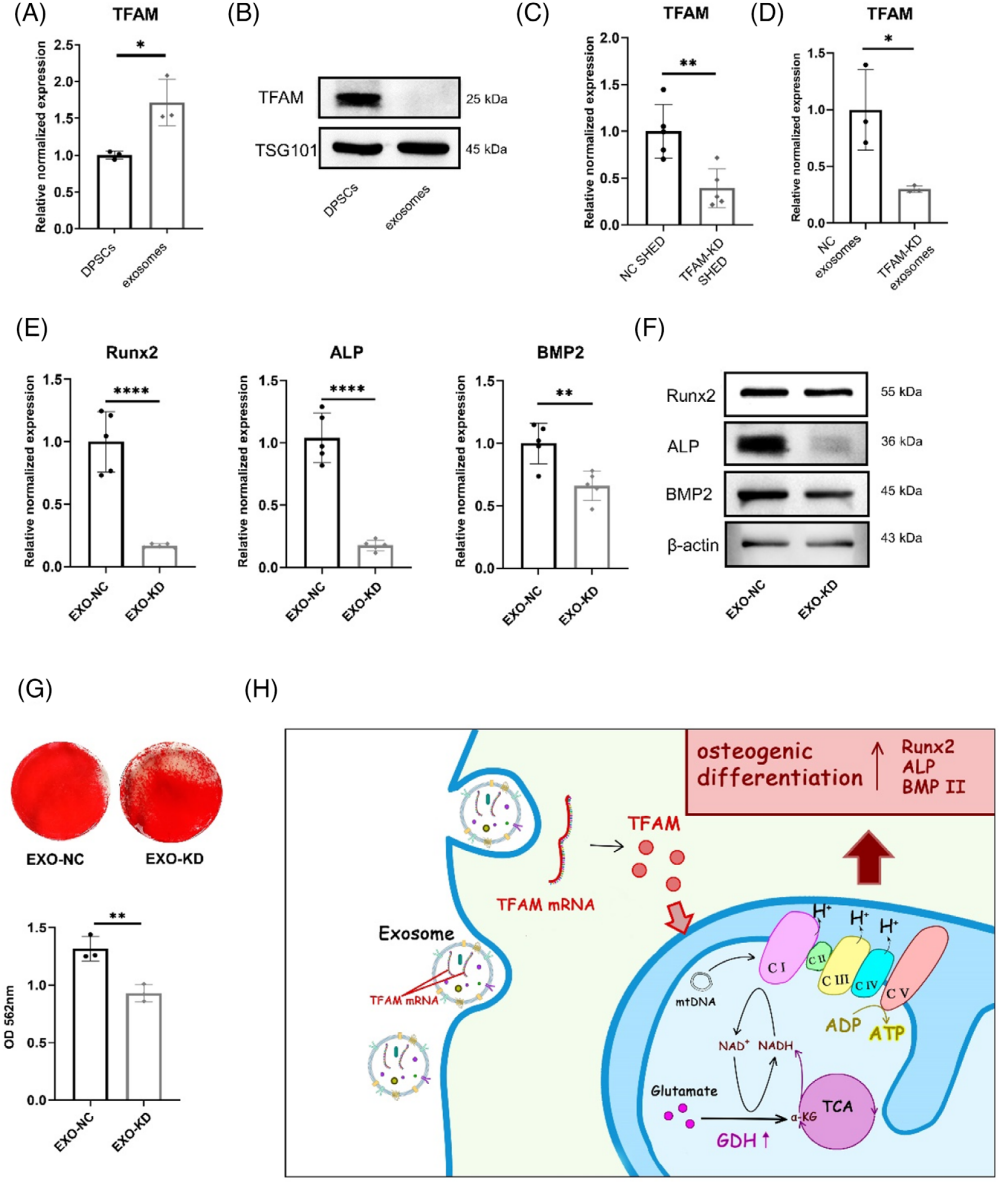
Exosomes (EXO) transfer mitochondrial transcription factor A (TFAM) mRNA to enhance the osteogenic capacity of dental pulp stem cells (DPSCs). (A) Exosomes from stem cells from human exfoliated deciduous teeth (SHED) aggregates contain more TFAM mRNA than DPSCs by quantitative real‐time reverse transcriptase–polymerase chain reaction (qRT‐PCR) analysis. *N* = 3 independent experiments. (B) Exosomes from SHED aggregates contain no TFAM protein by western blot analysis. (C) The TFAM mRNA of the CON, NC, TFAM‐KD, and EXO + TFAM‐KD groups by qRT‐PCR analysis. *N* = 5 independent experiments. (D) Exosomes from TFAM‐KD SHED (TFAM‐KD exosomes) contain less TFAM mRNA than Exosomes from NC SHED (NC exosomes) by qRT‐PCR analysis. *N* = 3 independent experiments. (E–G). DPSCs treated with TFAM‐KD exosomes show less osteogenesis capacity than with NC exosomes by qRT‐PCR analysis (E) (*N* = 5 independent experiments), western blot analysis (F), alizarin red staining, and quantitative analysis of the calcium deposition in DPSCs (G) (*N* = 3 independent experiments). (H) Graphic Summary shows that TFAM mRNA shuttled by exosomes promotes osteogenic properties of DPSCs through mitochondrial oxidative phosphorylation (OXPHOS) activation. *p < 0.05; **p < 0.01. Error bars are mean ± SD.

We then investigated the underlying mechanism by which exosomes increase the TFAM expression of DPSCs. Exosomes contained more TFAM mRNA than DPSCs (Figure 6A) but had no TFAM protein (Figure 6B). Then, we obtained TFAM‐KD SHED (Figure 6C) to isolate TFAM‐KD exosomes (Figure 6D). Next, exosomes from TFAM‐KD SHED (EXO‐KD group) and NC SHED (EXO‐NC group) were added to DPSCs to detect their osteogenic capacity. The qRT‐PCR assay revealed lower mRNA levels of ALP, Runx2 and BMP2 in the EXO‐KD group than in the EXO‐NC group (Figure 6E). Consistently, the EXO‐KD group expressed much less osteogenic‐related proteins, including ALP, Runx2 and BMP2, than the EXO‐NC group (Figure 6F, S3E). The alizarin red staining showed that mineralization of the EXO‐KD group was reduced (Figure 6G).

In conclusion, TFAM mRNA was shuttled by exosomes and then expressed in DPSCs. TFAM directly enhanced the expression of mitochondrial OXPHOS complexes and supported glutamate metabolism to enhance mitochondrial OXPHOS. Aerobic metabolism mediated by TFAM promoted osteogenic differentiation of DPSCs (Figure 6H).

## 3 DISCUSSION

In this study, we found that the combination of DPSCs and exosomes repaired defective cranial bone in mice and mandibular bone in rats. Our study revealed a previously unrecognized mechanism of exosomes in transferring TFAM mRNA to regulate mtDNA and GDH expression, which enhances mitochondrial OXPHOS and osteogenic differentiation of DPSCs.

Bone tissue engineering offers a promising alternative of generating constructs composed of instructive biomaterials, including MSCs and exosomes, which could enhance the outcome of reconstructive treatments.^20^ Different sources of exosomes have different efficacies in bone regeneration. Stem cell‐derived exosomes can regulate the distribution of macrophages around the bone healing interface by inhibiting the polarization of macrophages in the proinflammatory direction, thereby promoting bone healing.^21^ Exosomes derived from aging MSCs can slow the process of fracture healing by inhibiting Smad5.^22^ Moreover, optimized osteoinductive exosomes can be immobilized in a hierarchical scaffold for bone repair through the Bmpr2/Acvr2b competitive receptor‐activated Smad pathway.^23^ In our previous study, exosomes derived from SHED aggregates promoted angiogenesis in dental pulp regeneration.^24^ In the present study, we found that exosomes derived from SHED aggregates strengthened the osteogenic properties of DPSCs, and significantly promoted bone regeneration based on HA as a scaffold.

Exosomes prompted the rise of TFAM, which aroused our interest. TFAM binds to mtDNA to regulate the transcription of subunits of the mitochondrial electronic respiration chain so as to affect aerobic respiration of mitochondria,^25^ which is consistent with our results. Suppression of TFAM has been reported to cause decreased mitochondrial activity and function, impaired mitochondrial respiration and restrained osteogenesis.^26^ In our study, TFAM‐enhanced mitochondrial OXPHOS and relied on the catabolism of glutamate to support mitochondrial aerobic respiration. Glutamate is oxidized and deaminated to α‐KG and NADH by GDH and then flows into the TCA cycle.^16^ Importantly, we found that the deletion of TFAM caused a decrease in GDH, suggesting that TFAM might support glutamate metabolites for the TCA cycle to enhance mitochondrial OXPHOS. There have been no other similar reports about this, and we will further investigate the underlying mechanisms by which TFAM affects GDH.

Mitochondrial metabolism is emerging as an instructive signal for cell fate programs,^27^ and activated oxidative respiration has been demonstrated to be crucial for differentiation.^28^ Inhibiting the entry of pyruvate into the TCA cycle reduces the OXPHOS and differentiation ability of MSCs, while supplementation with α‐KG into the TCA cycle can restore OXPHOS and differentiation capacity.^29^ Key enzymes that regulate chromatin (both DNA and histones) and protein modifications (i.e., acetylation and methylation) rely on mitochondrial metabolic intermediates as cofactors.^30^ Hence, mitochondrial metabolism is inextricably coupled to gene expression and even regulates stem cell fate decisions.^31^ α‐KG subsequently upregulates BMP signalling through the decrease in H3K9me3 and H3K27me3.^32^, ^33^

We found that TFAM was necessary for osteogenesis differentiation in DPSCs. It has been reported that probiotic treatment increases TFAM expression in osteoblasts by promoting Kdm6b/Jmjd3 histone demethylase, which inhibits H3K27me3 epigenetic methylation at the TFAM promoter.^34^ Furthermore, TFAM‐transgenic mice fed a high‐fat diet did not experience an obesity‐linked reduction in glucose uptake, mitochondrial biogenesis and mineralization in osteoblasts.^34^ Moreover, we found that the knockdown of TFAM caused a decrease in GDH expression, but the mechanism needs to be further explored. Generally, exosomes could induce osteogenic differentiation by using their cargos.^35^ In our study, exosomes were rich in TFAM mRNA but did not contain TFAM protein compared with DPSCs. TFAM mRNA shuttled by exosomes was a probable element to promote osteogenic differentiation of DPSCs. Cargo nucleic acids in exosomes could be selectively carried by specific nucleotide sequences or RNA‐binding proteins.^36^ Recent studies have shown that stem cell‐derived exosomes can restore damaged OXPHOS processes by the transfer of TFAM mRNA, thereby repairing mitochondrial damage to some extent and exerting anti‐inflammatory effects.^37^

## 4 MATERIALS AND METHODS

### 4.1 Cell isolation

The experimental protocols were approved by the Hospital Ethics Committee (No. IRB‐REV‐2018020). Consent forms were obtained before conducting this research project. Tooth samples from healthy adults and kids were collected from 7 adults, aged 20–30 years and 3 kids, aged 8–10 years. Donor information is listed in Tables S2 and S3. Teeth were extracted at the Department of Oral and Maxillofacial Surgery, School of Stomatology, Fourth Military Medical University. Briefly, the dental pulp was cut to a size of 1 mm and enzymatically digested with 3 mg/ml type I collagenase (SCR103, Sigma‐Aldrich, USA) for 2 h at 37°C (suspended every 15 min). Cells were then plated in 6‐well plates in alpha modification of Eagle's medium (α‐MEM); (11900, Gibco, USA) with 10% fetal bovine serum (FBS) (11011–8611, Tianhang, China), 0.292 mg/ml l‐glutamine (25030081, Invitrogen, USA), 100 units/ml penicillin and 100 mg/ml streptomycin (15140163, Gibco, USA) at 37°C in 5% CO_2_, cultured for 2 weeks and the medium was changed every 3 days. To further purify the stem cells, single‐cell‐derived colonies were obtained using the limiting dilution technique.

SHED was isolated and cultivated under the protocol previously described. SHED used for each experiment was at passage 2nd–5th.

### 4.2 Exosomes uptake assay

Enriched exosomes were labelled with PKH67 (MID167, Sigma) in accordance with the manufacturer's protocol. DPSCs were seeded in confocal dishes (Nunc, USA) and incubated with the labelled exosomes for 24 h. 4′,6‐diamidino‐2‐phenylindole (1:50, 190305, MP Biomedicals) was used for staining nuclei, and MitoTracker (MP07510, Invitrogen, USA) was used for staining mitochondria. The uptake of exosomes was visualized by a confocal fluorescence microscope (Nikon, Japan).

### 4.3 qRT‐PCR analysis

Total RNA was extracted with TRIzol reagent (15,596,026, Invitrogen) and converted to cDNA using PrimeScript™ RT Master Mix Kit (RR036A, Takara, Japan). Then, qRT‐PCR was conducted with TB Green® Premix Ex Taq™ II (RB820A, Takara) using the quantitative PCR System (Bio‐Rad, USA). The primers are shown in Table S1.

### 4.4 Western blot

For osteogenic experiments, the osteogenic medium containing 90 μg/ml exosomes or PBS was changed every 48 h. For metabolism experiments, exosomes were added to the growth medium every 48 h for a total of 2 times.

Primary antibodies employed in this study included β‐actin (1:1000, CW0096A, CWBIO), ALP (1:500, sc‐79840, Santa Cruz Biotechnology), Runx2 (1:1000, #12556, Cell Signalling), BMP2 (1:1000, ab14933, Abcam), TFAM (1:1000, sc‐376,672, Santa Cruz Biotechnology), total OXPHOS cocktail (1:1000, ab110413, Abcam), Glut1 (1:1000, er1510‐11, Huabio), GDH (1:1000, 12793S, Cell Signalling), and CPT1A (1:1000, 12252S, Cell Signalling). The secondary antibody was HRP‐conjugated to antibodies in rabbits and mice. The amount of protein was semiquantified by grey‐scale analysis using ImageJ 1.53c (National Institutes of Health, USA) and normalized to β‐actin as an internal standard.

### 4.5 Alizarin red staining

Alizarin red staining (Sigma, USA) was used to assess calcium deposits, and 10% cetylpyridinium chloride was added for quantitative analysis. The absorbance values were measured at 562 nm.

### 4.6 Cranial bone defect model

Six‐week‐old male C57BL/6J mice for the cranial bone defect model were purchased from the Animal Center of the Fourth Military Medical University, China. All animal experiments were approved by the Animal Ethics Committee of the Fourth Military Medical University, Xi'an, China (No. 2022030).

The cranial bone defect model used was modified based on the previous research.^38^ The diameter of the defect area was about 2.5 mm. A total of 24 mice were randomly divided into four groups: (1) a control group without cell and HA powder (677418, Sigma) implantation (control, *n* = 6); (2) a group with 20 mg HA powder only (HA, *n* = 6); (3) a group treated with DPSC aggregates (5 × 10^5^ DPSCs were seeded in 6‐well‐plate and induced to aggregates under medium containing 50 mg/ml vitamin C after 7 days, and the number of DPSC aggregates in a well was about 5 × 10^6^) wrapping 20 mg HA powder (DPSCs + HA, *n* = 6); and (4) a group treated with the combination of DPSC aggregates and exosomes (90 μg/ml exosomes were added into DPSCs every 48 h during the aggregates' induction), wrapping 20 mg HA powder (EXO + DPSCs + HA, *n* = 6). After 8 weeks, the samples were harvested from the mice and fixed with 4% paraformaldehyde for 12 h.

### 4.7 Micro‐CT analysis

Bone samples were scanned using a Micron X‐ray three‐dimensional (3D) Imaging System (YXLON, Germany) with 9 μm resolution. 3D images were reconstructed and analysed by the VG Studio 3.4 (VG, Germany). For measurement, a region of interest (ROI) of approximately 2.5 mm in diameter was defined in the cranial bone. The BV/TV, the ratio of new bone area to the defect area, Tb.N and Tb.Sp were calculated.

### 4.8 Histological analysis

After micro‐CT analysis, the mandibles were decalcified with 17% ethylenediaminetetraacetic acid (EDTA) (EDTA0500, MP Biomedicals) for 1 month and then embedded in paraffin. Paraffin sections (3‐mm‐thick) were stained using HE, as described previously.^39^ The percentage of new bone area to the total area was evaluated quantitatively from three randomly‐selected sections by ImageJ 1.53c.

### 4.9 Masson trichrome staining

The paraffin sections were stained with Masson trichrome staining (4079A, Baso Diagnostic Inc., China) in accordance with the manufacturer's instructions. Photographs were taken using a microscope (OLYMPUS).

### 4.10 Seahorse metabolic flux analysis

DPSCs (2 × 10^4^/well) were seeded in XF24‐cell culture plates (Agilent, USA) and treated with exosomes for 24 h under the growth conditions. The OCR and ECAR were measured in an XF24 Flux Analyser (Seahorse Bioscience, USA). In the OCR assay, cells were first measured under basal conditions and then stimulated with oligomycin (1 μM), FCCP (2 μM), rotenone (2 μM) and antimycin A (2 μM) to determine different parameters of mitochondrial functions. In the ECAR assay, glucose (10 mM), oligomycin (1 μM) and 2‐DG (50 mM) were subsequently added into the medium.

### 4.11 NADH/NAD assay

DPSCs (10^6^/well) treated with exosomes at 0, 24, 48 and 72 h were detected by NADH/NAD assay kit (S0175, Beyotime, China) in line with the instructions.

### 4.12 ATP assay

A total of 10^5^ DPSCs were collected, resuspended with 100 μl CellTiter‐Glo® 2.0 Reagent (Promega, America), and transferred to a 96‐well plate. The 96‐well plate was placed on a micro‐shaker for 2 min, incubated at room temperature while avoiding light for 10 min, and its chemiluminescence value was detected.

### 4.13 shRNA‐mediated knockdown of TFAM in DPSCs and SHED

For the shRNA knockdown experiments, DPSCs and SHED were treated with lentivirus, which carried TFAM shRNAs, in line with the manufacturer's protocol (Shanghai Genechem Co., Ltd.). Briefly, DPSCs and SHED were infected with shTFAM lentivirus at a multiplicity of infection (MOI) of 10. At 48 h after the shRNA induction, the cells were analysed for expression levels of TFAM and were used for the following experiments.

### 4.14 Statistical analysis

All results were presented as the mean ± SD of at least three independent experiments. Two‐group comparisons were analysed by Student's *t* tests. Comparisons among three or four groups were evaluated by one‐way ANOVA followed by an LSD post hoc test. A *p*‐value less than 0.05 was considered statistically significant.

## 5 CONCLUSION

Exosomes derived from SHED aggregates promote DPSCs osteogenic differentiation, which contribute to cranial bone defect reparation. Moreover, the exosomes transfer TFAM mRNA to DPSCs and might activate mitochondrial OXPHOS to increase osteogenic differentiation. Our study provides a new potential strategy to improve the clinical therapy with DPSCs in bone regenerative medicine.

## AUTHOR CONTRIBUTIONS

Jia Guo contributed to the study design, execution, data acquisition, and interpretation. Feng Zhou and Yuan Cao performed the animal experiments. Zhi Liu and Qiming Zhai helped in cell collection and data analysis. Wanming Zhao contributed to study design and interpretation. Zheru Zhang drew the picture. Bei Li, Fang Jin and Yan Jin designed the experiments, oversaw the collection of results and data interpretation, and drafted the reports. All authors have seen and approved the final version.

## CONFLICT OF INTEREST

The authors have declared that no competing interest exists.

## Supporting information


**Appendix S1** Supporting Information.

## Data Availability

Research data are not shared.
