# Supplementary material for: Exosome‐shuttled mitochondrial transcription factor A mRNA promotes the osteogenesis of dental pulp stem cells through mitochondrial oxidative phosphorylation activation
Source: Cell Prolif. 2022 Aug 26;55(12):e13324. doi: 10.1111/cpr.13324 (PMC9715363; doi:10.1111/cpr.13324)
Supplement: Supplementary file 1 — Appendix S1 Supporting Information. [file CPR-55-e13324-s001.docx]

**Supplemental Materials**

**
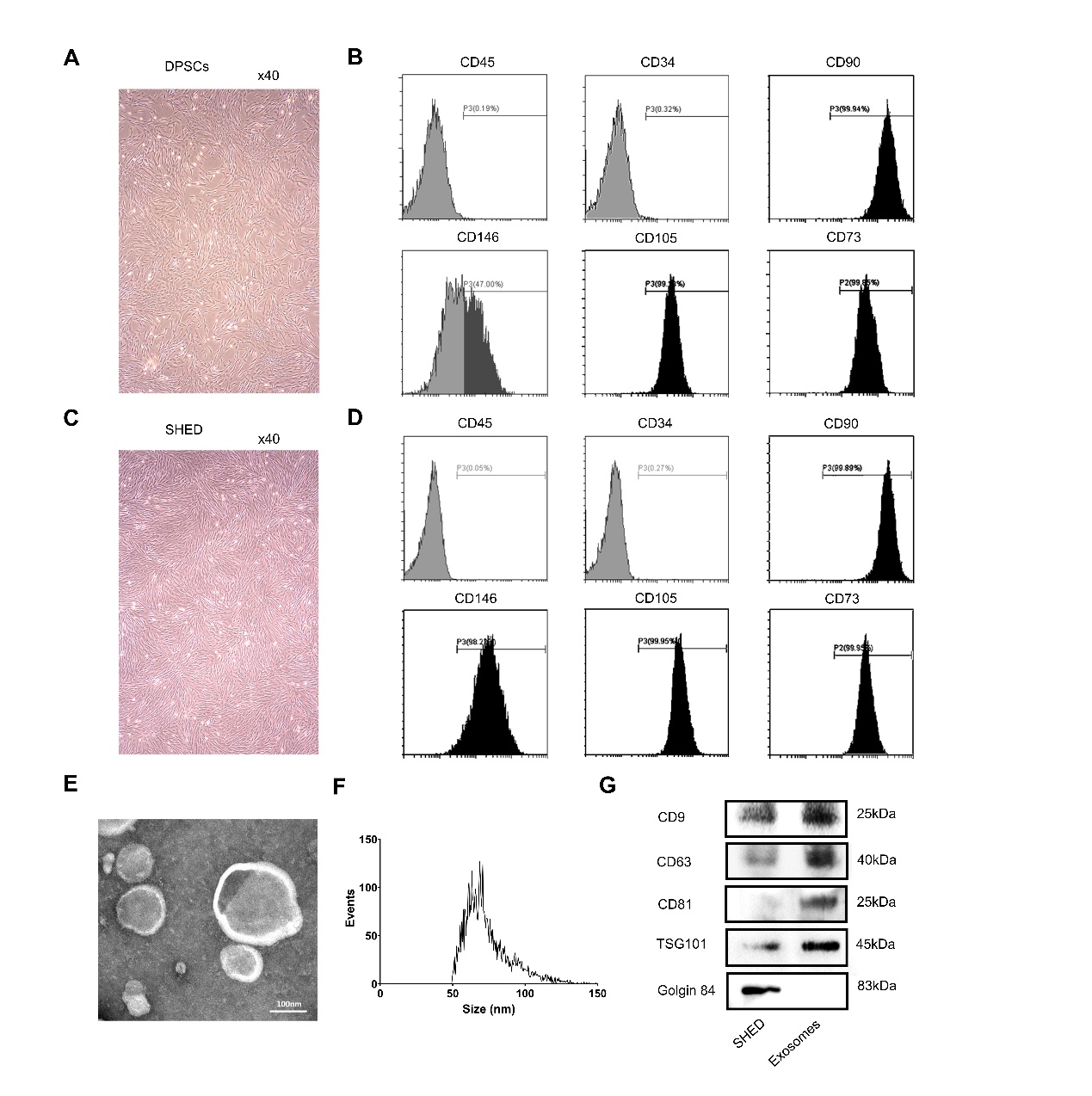
Fig. S1 Characterization of exosomes**

A. The morphology of DPSCs is observed under the 40X microscope. B. Surface markers (CD34, CD45, CD90, CD146, CD105, and CD73) of DPSCs are detected by Flow cytometry. C. The morphology of SHED is observed under the 40X microscope. D. Surface markers (CD34, CD45, CD90, CD146, CD105, and CD73) of SHED are detected by Flow cytometry. E. TEM observation of the morphology of exosomes. Scale bar: 100 nm. F. Particle size distribution and concentration of exosomes measured by NTA. G. Western blot analysis of the exosomal surface markers (CD9, CD63, CD81, and TSG101) and a negative marker (Golgin84).


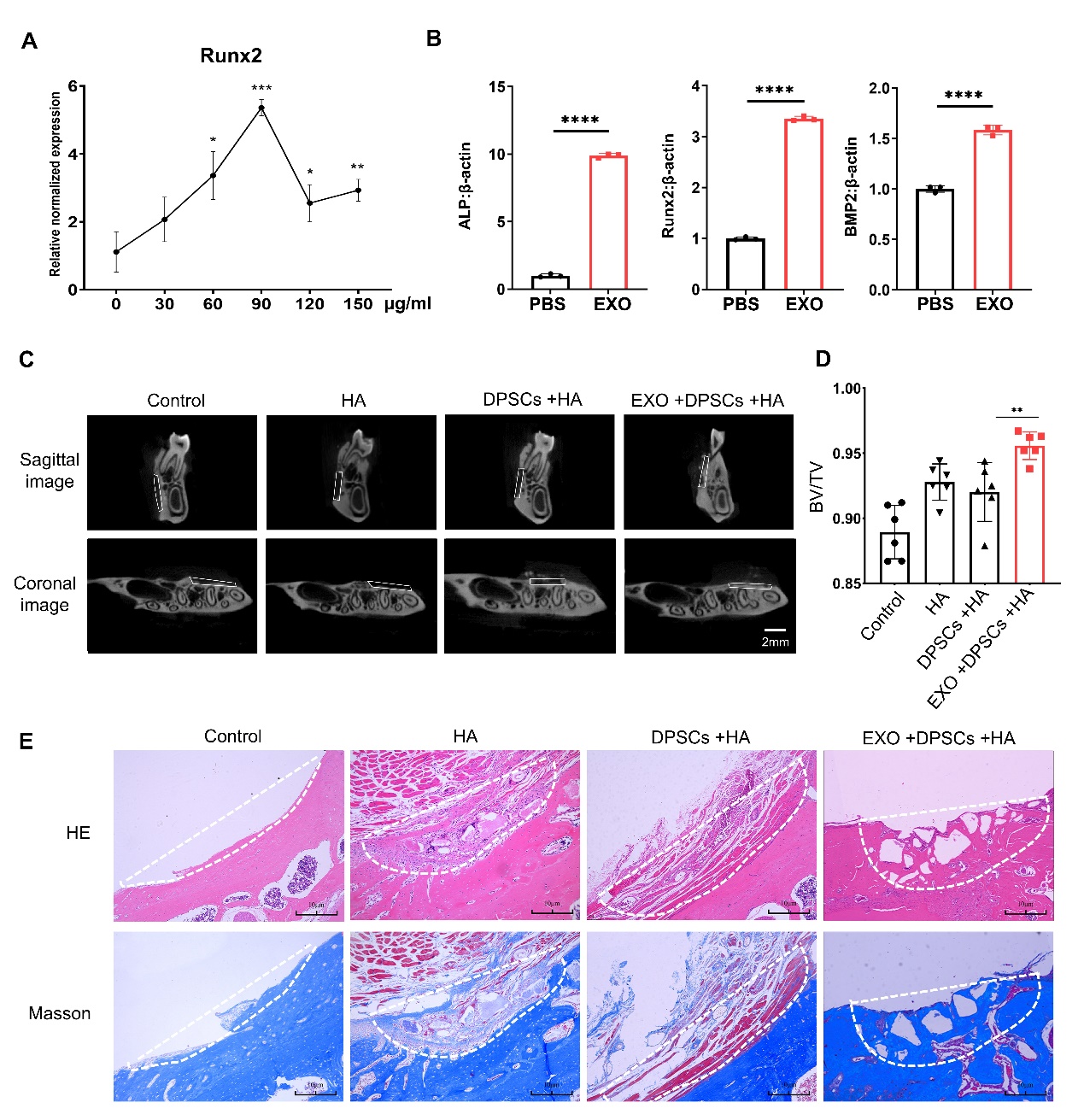


**Fig. S2 Exosomes with DPSCs promote bone regeneration of mandibular bone**

A. The expression of Runx2 in DPSCs treated with gradient concentrations of exosomes (0, 30, 60, 90, 120, 150 μg/ml). N = 5 independent experiments. B. Quantitative analysis of western blot analysis in the PBS and EXO group. N = 3 independent experiments. C. micro-CT sagittal and coronal section images of the Control, HA, DPSCs+ HA, EXO+ DPSCs+ HA groups. The mandibular bone defect area was marked with a white line. Scale bar: 2 mm. D. Quantitative analysis of bone volume/total volume. N = 6 independent examples. E. HE staining and Masson trichrome staining of sagittal sections. Scale bar: 10 μm. **P*<0.05; ***P*< 0.01, ****P*< 0.001, *****P*< 0.0001. Error bars are mean ± SD.


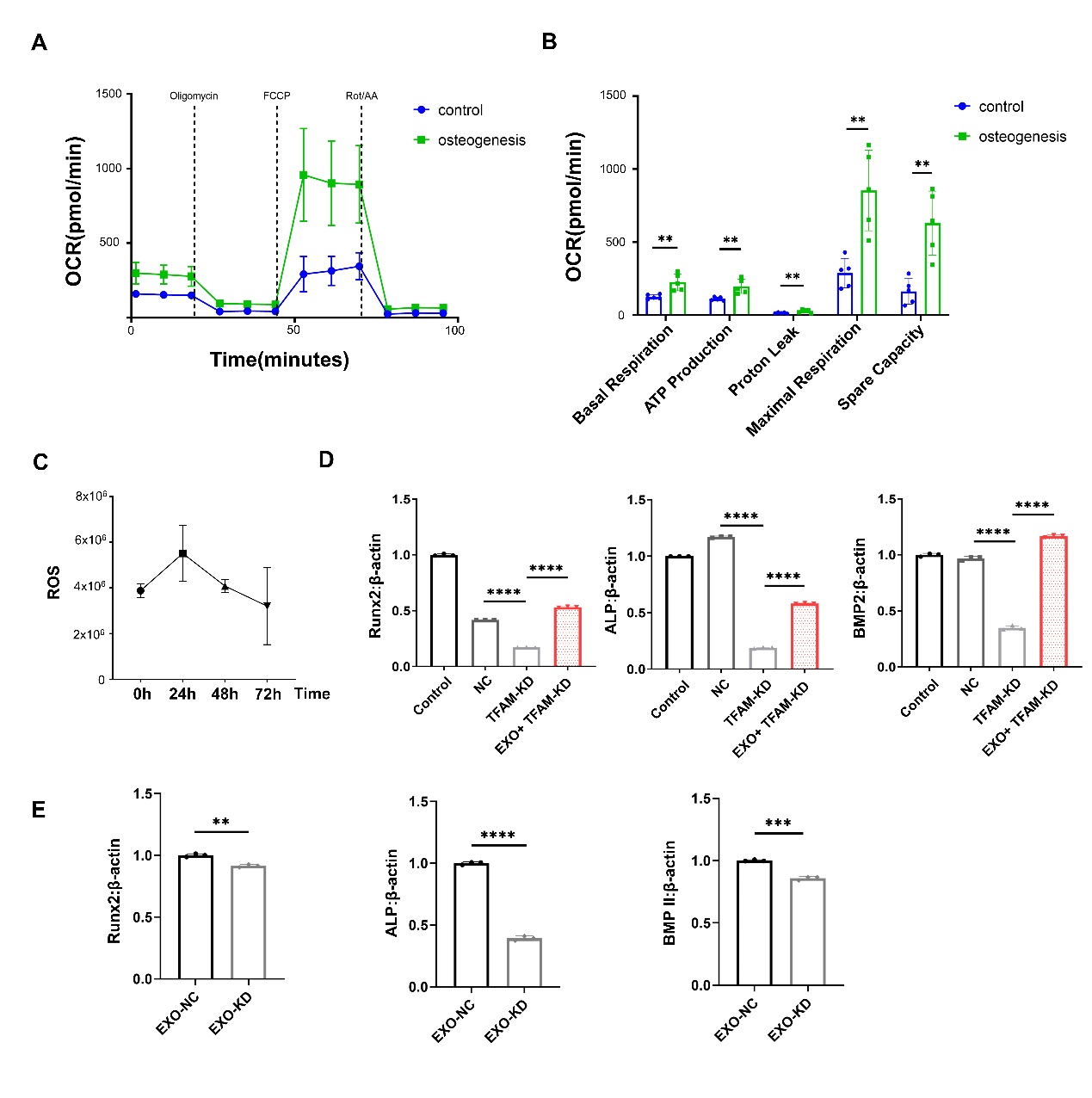


**Fig. S3 OCR of DPSCs after osteogenic induction and redox statement of DPSCs with exosomes for 3days.**

A. OCR from seahorse analysis in DPSCs after osteogenic induction. B. Basal respiration, ATP production, proton leak, maximal respiration, and spare capacity in OCR assay. N = 5 independent experiments. C. ROS of DPSCs with exosomes for 72 hours. N = 3 independent experiments. D. Quantitative analysis of western blot analysis in the Control, NC, TFAM-KD, EXO+TFAM-KD group. N = 3 independent experiments. E. Quantitative analysis of western blot analysis in the EXO-NC and EXO-KD group. N = 3 independent experiments. **P*< 0.05. Error bars are mean ± SD, ***P*< 0.01, ****P*< 0.001, *****P*< 0.0001. Error bars are mean ± SD.

**Table S1. Sequences of primers of PCR product**

| Gene name | Sequence (5'→3') |
| --- | --- |
| β-actin | F：TGGCACCCAGCACAATGAA |
|  | R：CTAAGTCATAGTCCGCCTAGAAGCA |
| Runx2 | F：CGGAATGCCTCTGCTGTTATG |
|  | R：AAGGTGAAACTCTTGCCTCGTC |
| ALP | F：GTGAACCGCAACTGGTACTC |
|  | R：GAGCTGCGTAGCGATGTCC |
| BMP2 | F：GACTGCGGTCTCCTAAAGGTCG |
|  | R：CTGGGGAAGCAGCAACGCTA |
| TFAM | F：AGCTCAGAACCCAGATGCAA |
|  | R：CCGCCCTATAAGCATCTTGA |

Runx2: Runt-related transcription factor 2; ALP: alkaline phosphatase; BMP2: bone morphogenetic protein 2; TFAM: mitochondrial transcription factor A.

**Table S2. Donor information of DPSCs**

| Cells | Age | Gender |
| --- | --- | --- |
| DPSCs1 | 24 | Male |
| DPSCs2 | 29 | Female |
| DPSCs3 | 26 | Male |
| DPSCs4 | 30 | Female |
| DPSCs5 | 30 | Male |
| DPSCs6 | 23 | Female |
| DPSCs7 | 25 | Female |

DPSCs: dental pulp stem cells.

**Table S3. Donor information of SHED**

| Cells | Age | Gender |
| --- | --- | --- |
| SHED1 | 8 | Male |
| SHED2 | 8 | Female |
| SHED3 | 10 | Female |

SHED: stem cells from human exfoliated deciduous teeth.

**Supplemental Methods**

**Flow cytometry**

To identify DPSCs and SHED, CD34, CD45, CD90, CD73, and CD105 (Biolegend, USA) were chosen as markers and measured using flow cytometric analysis (Cytoflex, Beckman Coulter, CA, USA).

**Exosomes Isolation**

SHED (5 × 10^5^ per well) were seeded in a 6-well-plate with basal medium and changed for aggregates induction medium (basal medium supplemented with 50 mg/ml vitamin C (100769, MP Biomedicals, USA). The induction medium was refreshed every 3-4 days. The aggregate was harvested after 10 days for the following experiments.

Then the SHED aggregates were cultured in the medium with exosome-deleted FBS for 48 h. Exosomes-deleted FBS was prepared by ultracentrifuging for 16 h at 100,000 g before use. Finally, the supernatants were collected and several centrifugates were performed for exosome isolation as described previously^1^. Briefly, the supernatants were centrifuged at 300 g for 10 min, 2000 g for 10 min, and 16,000 g for 30 min to remove dead cells and cellular debris, respectively. Then, the supernatants were ultra-centrifuged at 150,000 g for 70 min and additionally washed with PBS for another 70 min at 150,000 g. All procedures were performed at 4 °C. The final pellets were resuspended in sterile PBS and stored at -80 °C for the following experiments.

**Characterization of Exosomes**

Western blotting was performed to detected exosome markers, including TSG101 (1:1000, ab125011, Abcam, USA), CD81 (1:1000, sc-166029, Santa Cruz-Biotechnology, USA), CD9 (1:1000, ab92726, Abcam), Golgin84 (1:1000, ab155806, Abcam). Cell extract was considered as a control. Transmission electron microscopy (TEM) (Thermo Fisher, USA) was conducted to observe the morphology of exosomes. The exosomes were dropped onto carbon-coated copper grids and kept for 3-4 min, then dried with filter paper and stained with 1% phosphotungstic acid for 10 s, finally washed with distilled water for 30 s. Nanoparticle tracking analysis (NTA) was utilized to identify the particle size and distribution.

**Mandibular Bone Defect Model**

Six-week-old male Sprague-Dawley (SD) rats for the mandibular bone defect model were purchased from the Animal Center of the Fourth Military Medical University, China. The surgical procedures were approved by the Animal Ethics Committee of the Fourth Military Medical University, Xi’an, China (No.2021046). The mandibular bone defect model used was modified based on the previous research^2^. A mandibular bone defect of approximately 3 × 2 × 1 mm^3^ was created at the buccal mandibular bone of the left molars. A total of 24 SD rats were randomly divided into 4 groups as previously described: (1) control, n = 6 independent examples; (2) HA, n = 6 independent examples; (3) DPSCs+HA, n = 6 independent examples; and (4) EXO+DPSCs+HA, n = 6 independent examples. After 4 weeks, the rats’ mandible samples were harvested and fixed with 4% paraformaldehyde (158127, sigma) for 48 h.

For measurement, a region of interest (ROI) of approximately 3 × 2 × 1 mm^3^ was defined, at buccal mandibular bone from first to second mandibular molars. The ratio of new bone volume to tissue volume (BV/TV) was calculated.

**ROS Assay**

DPSCs (10^6^ /well) treated with exosomes at 0 h, 24 h, 48 h, and 72 h were detected by ROS assay Kit (S0033, Beyotime, China) according to the instructions.

**Reference**

1. Théry C, Amigorena S, Raposo G, et al. Isolation and characterization of exosomes from cell culture supernatants and biological fluids. Current protocols in cell biology. 2006;30(1):1-29.

2. Sun J, Dong Z, Zhang Y, et al. Osthole improves function of periodontitis periodontal ligament stem cells via epigenetic modification in cell sheets engineering. Sci Rep. 2017;7(1):5254.
